# Late evaluation of the relationship between morphological and functional renal changes and hypertension after non-operative treatment of high-grade renal injuries

**DOI:** 10.1186/1749-7922-7-26

**Published:** 2012-08-01

**Authors:** Gerson Alves Pereira Júnior, Valdair Francisco Muglia, Antônio Carlos dos Santos, Cecilia Hissae Miyake, Fernando Nobre, Mery Kato, Marcus Vinicius Simões, José Ivan de Andrade

**Affiliations:** 1 Department of Surgery and Anatomy, Division of Trauma and Emergency Surgery, University of São Paulo, Sao Paulo, Brazil; 2 Department of Internal Medicine, University of São Paulo, Sao Paulo, Brazil; 3 Image and Medical Physics Center Division, University of São Paulo, Sao Paulo, Brazil; 4 Cardiology Division; Faculty of Medicine of Ribeirão Preto, University of São Paulo, Sao Paulo, Brazil; 5 Hospital das Clinicas da Faculdade de Medicina de Ribeirão Preto – USP, Unidade de Emergência, Centro de Estudos de Emergências em SaúdeRua Bernardino de Campos, 1000, CEP 14015-130 Ribeirão Preto, São Paulo, Brasil

**Keywords:** Renal injury, Conservative management, Follow-up imaging, Renal function, Radionuclide imaging, Dimercaptosuccinic acid, Computed tomography, Magnetic resonance angiography, Renal hypertension

## Abstract

**Objective:**

To evaluate the anatomical and functional renal alterations and the association with post-traumatic arterial hypertension.

**Methods:**

The studied population included patients who sustained high grades renal injury (grades III to V) successfully non-operative management after staging by computed tomography over a 16-year period. Beyond the review of medical records, these patients were invited to the following protocol: clinical and laboratory evaluation, abdominal computed tomography, magnetic resonance angiography, DMSA renal scintigraphy, and ambulatory blood pressure monitoring. The hypertensive patients also were submitted to dynamic renal scintigraphy (^99m^Tc EC), using captopril stimulation to verify renal vascular etiology.

**Results:**

Of the 31 patients, there were thirteen grade III, sixteen grade IV (nine lacerations, and seven vascular lesions), and two grade V injuries. All the patients were asymptomatic and an average follow up post-injury of 6.4 years. None had abnormal BUN or seric creatinine. The percentage of renal volume reduction correlates with the severity as defined by OIS. There was no evidence of renal artery stenosis in Magnetic Resonance angiography (MRA). DMSA scanning demonstrated a decline in percentage of total renal function corresponding to injury severity (42.2 ± 5.5% for grade III, 35.3 ± 12.8% for grade IV, 13.5 ± 19.1 for grade V). Six patients (19.4%) had severe compromised function (< 30%). There was statistically significant difference in the decrease in renal function between parenchymal and vascular causes for grade IV injuries (p < 0.001). The 24-hour ambulatory blood pressure monitoring detected nine patients (29%) with post-traumatic hypertension. All the patients were male, mean 35.6 years, 77.8 % had a familial history of arterial hypertension, 66.7% had grade III renal injury, and average post-injury time was 7.8 years. Seven patients had negative captopril renography.

**Conclusions:**

Late results of renal function after conservative treatment of high grades renal injuries are favorable, except for patients with grades IV with vascular injuries and grade V renal injuries. Moreover, arterial hypertension does not correlate with the grade of renal injury or reduction of renal function.

## Introduction

The non-surgical management of high-grade renal injuries is initially successful in more than 85% of patients [1-3]. The Organ Injury Scale (OIS) of the American Association for the Surgery of Trauma (AAST) is of utmost clinical importance since the higher the renal injury grade with the higher the frequency of surgery [4].

The primary objective of the non-surgical treatment is to preserve enough renal parenchyma to prevent dialysis in the case of loss of the contralateral kidney (to achieve approximately 30% function of a normal kidney) [5-9].

There has long been interest in quantitative dimercaptosuccinic acid (DMSA) renal scintigraphy for long-term evaluation of renal function after trauma and surgery. In spite of some series recently published, usually post-injury follow-up is and evaluation of kidney function were inadequate in the literature [1,10-15].

Arterial hypertension is an uncommon complication of renal trauma, although reports on its incidence vary from 1 to 40% [16-19]. Despite the relative scarcity of this complication, its potential negative impact on life expectancy and morbidity makes a serious complication [18,20]. Posttraumatic renovascular hypertension is usually renin dependent, and associated with vascular and renal parenchymal injury [18,20]. Captopril renography is a useful and reliable test in patients with suspicion of renovascular hypertension [21,22].

In this study, we aimed to follow patients with high grades (grades III, IV e V) renal injuries after successfully non-operative management. This late evaluation should establish the degree of functional deficit of the injured kidney, its clinical and laboratorial repercussions and also the incidence and etiology of the arterial hypertension arising after trauma, to verify if it is essential or renovascular origin.

## Materials and methods

After approval from the Research Ethics Committee, we retrospectively reviewed the patients with renal injuries over a 16-year period, including all patients who had high grades renal injury (grades III to V) successfully non-operative management after staging by computed tomography between January 1989 and December 2004. Non-operative treatment included bed rest, close clinical observation with monitoring of vital signs and serial haematocrit studies. Except in three patients, intravenous antibiotic was given during hospital stay. Patients with gross haematuria were kept on bed rest until the urine was clear.

The medical records were reviewed for patient age, injury mechanism, injury side, significant associated abdominal injuries, past medical history, physical findings including macroscopic hematuria, laboratorial findings, radiological imaging, medical and surgical management, blood transfusion requirements, length of hospital stay, and the development of urological complications. CT images were graded according to the renal injury scale recommended by the Organ Injury Scaling Committee of the American Association for Surgery of Trauma (AAST) and revised by a staff radiologist.

In addition of medical records reviewing, these patients were invited to entry in a follow-up research protocol. The post-trauma follow-up goals were: 1) to clinically evaluate patients, regarding complaints, past medical history, family history, and findings in the physical examination, 2) to evaluate kidney morphology and the renal blood flow by means of computed tomography of abdomen and MRA, 3) to evaluate renal function by using DMSA renal scintigraphy to detect and quantify differences in renal function, 4) to evaluate the incidence of arterial hypertension in the follow-up of these cases by using ambulatory blood-pressure monitoring, 5) to evaluate if anatomical and functional kidneys alterations in association with arterial hypertension correlate with the grade of renal trauma, defined by CT, at the patient’s admission and 6) when hypertension were present, to investigate possible renal vascular etiology by dynamic ^99m^technetium ethylenedicysteine (^99m^Tc EC) renal scintigraphy, using the captopril-stimulated study.

For laboratory evaluation, all patients of the study had: serum levels of urea and creatinine, electrolytes (sodium, potassium and calcium), total protein, albumin, lipidogram (cholesterol, LDL, HDL and triglycerides), hemoglobin, hematocrit, fasting glycemia and urine analysis.

Abdominal CT scans were performed also, to detect and monitor complete resolution of perinephric hematoma and urinoma, when present.

Magnetic resonance were performed on a 1.5 Tesla scanner, Magneton Vision, from Siemens (Erlangen – Germany), with a dedicate torso coil. We employed sequences to evaluate renal morphology and the status of major renal arteries. Our protocol includes images weighted in T1 and T2, on axial and coronal planes, using Gradient-Echo and Turbo Spin-Echo sequences. For MRA, we used the “bolus test” technique to set the ideal time for the arterial phase. A 3D-Gradient-Eco sequence was applied along the coronal plane for angiography (Repetition Time = 4.6 ms and Echo Time =1.8 ms, flip angle of 25 degrees and 1.0 mm slice thickness). Images were processed at a Siemens workstation using Maximum Intensity Projection (MIP) and Multiplanar Reformatting (MPR) techniques for angiography. Flow quantification was performed using phase-contrast sequence (TR = 24.0 ms, TE = 5.0 ms, Flip Angle = 30) with cardiac and respiratory gating. Flow measurements were also performed at the same workstation using the software Flow Quantification provided by Siemens Medical Systems. Peak systolic velocity and acceleration time were the additional hemodynamic parameters evaluated.

Quantitative DMSA scintigraphy was performed in all patients. Differential renal function was calculated by adding the individual counts of both kidneys and recording the fractional contribution of each kidney as a percentage of total renal function. Relative renal function as viewed by DMSA renal scintigraphy was standardized as follows: normal to mild impairment if the injured kidney achieved more than 40% differential renal function; moderate impairment if the injured kidney provided 30-40% differential function; and severe if the injured kidney contributed less than 30% [10].

The ambulatory blood pressure monitoring was programmed to take measurements every 15 to 30 minutes throughout the day and night, respectively. The oscillometric technique utilized a Spacelabs 90207 monitor (Spacelabs Medical Inc, Issaquah, WA, USA). The upper limit of normality for daytime ambulatory blood pressure was defined as 135/85 mmHg.

In hypertensive patients, Captopril-stimulated study was performed after baseline assessment with ^99m^Tc EC scintigraphy and 1 hour after oral administration of 25 mg.

Statistical analysis was performed with StatView® using analysis of variance (Anova) or the test of Kruskal – Wallis. The IC was set to 95% and significance was considered at p <0,05.

## Results

A total of 66 patients were admitted with high grades renal injury (grades III to V) secondary to trauma. All patients were successfully assessed with non-operative management after tomographic staging. Of these 66 patients, 31 of them agreed to be included in the study and were submitted to clinical, laboratorial, morphological and functional studies.

Of 31 patients with renal trauma successfully treated conservatively, the median age was 23.9 years at the time of admission (range 4 – 60 years). Patient gender, AAST renal injury grade, side of injury and presence of gross haematuria are listed in Table 1. Blunt trauma occurred in 27 (87.1%) cases: motor vehicle accident (8), motorcycle accident (7), pedestrian struck (3), falls (5), animal related accident (3) and assault (1). Of the 4 penetrating traumas (12.9%): stab wounds (2) and gunshot wounds (2).

**Table 1:** Patients characteristics at the admission

	**N (%)**
Gender:	
Female	6 (19.4)
Male	25 (80.6)
Age:	
Younger than 18	9 (29)
18 or greater	22 (71)
Renal Trauma Grade:	
III	13 (41.9)
IV:	16 (51.6)
IV p	(parenchymal) 9 (29)
IV v	(vascular) 7 (22.6)
V	2 (6.5)
Side:	
Left	15 (48.4)
Right	16 (51.6)
Gross haematuria:	
No	2 (6.5)
Yes	29 (93.5)

Only 5 patients required blood transfusion (16.1%), a total of 4090 ml. Of these, 4 (80%) had grade IV renal trauma with vascular injury. All patients had normal serum creatinine at admission. The length of hospital stay varied from 2 to 27 days, and averaged 7.8 days.

The time elapsed from admission for renal trauma to the initial follow-up varied from 1 year and 4 months to 14 years and 5 months, averaging 6 years and 4 months, as shown in Table 2. There was no significant difference among the grades of renal trauma.

**Table 2:** Severity of the renal injuries and mean follow-up times

**Grade of injury**	**Numbers of cases**	**Mean follow-up time**
III	13	7y6m
IV p	9	7y3m
IV v	7	4y7m
V p	1	1y6m
V v	1	1y4m
Total	31	

At the clinical evaluation, all the patients were asymptomatic while one patient referenced arterial hypertension and 41.9% of them related family history of arterial hypertension. There was no alteration detected in the physical examination. Body mass index was greater than 25 Kg/m [2] in 41.9% of the patients.

Levels of serum blood-urea-nitrogen, creatinine, sodium, potassium, calcium, glycemia, albumin, total proteins, hemoglobin as well as the white cell count were within normal limits. Furthermore, no alterations were found in the urine analysis. In relation to the lipid panel, 6 patients (19.4%) had serum cholesterol levels greater than 200 mg/dl and 3 of them also had elevated triglyceride levels greater than 150 mg/dl. Another 7 patients had isolated hypertriglyceridemia (22.6%).

Regarding the tomographic evaluation, patients with grade III renal trauma showed decreased volume of the injured kidney in 23.1% of the cases (3); 44.4% (4) were grade IV cases with contrast extravasation and 85.7% (6) had grade IV renal trauma with vascular injury; both patients with renal trauma grade V showed diminished kidney parenchyma (100%). The Kruskal-Wallis test showed significant difference between grade III and grade IV with pedicle injury.

The MRA of all patients of the study showed no renal artery stenosis. Flow quantification was complete in 23 patients (74.2%) with measurements considered adequate for the analysis. Quantitative blood flow differences between the two kidneys were measured to provide comparisons in percentages of flow reduction between the sides. Asymmetry of blood flow were considered relevant when higher than 15% [23-26].

The blood flow asymmetry found between the two kidneys was higher than 15% in 91.3% of the patients (21 in 23 cases). Results showed eleven patients with grade III renal trauma (78.6%) with average flow reduction of 42.7%; six patients (66.7%) with injury grade IV with extravasation showing an average reduction of 34.5%; five grade IV renal trauma patients (71.4%) with vascular injury reduced by an average of 50.1% and one patient with grade V renal injury with total kidney devascularization presenting a blood flow reduction of 86.5% on the injured side. The statistical analysis showed that, despite the high variation in percentage of blood flow reduction among the different grades of renal trauma, there was no significant difference among the groups. Table 3 summarizes the data of the CT and magnetic resonance angiography.

**Table 3:** Patients with reduction in renal volume tomography and average flow reduction in magnetic resonance angiography observed by grade renal injury

**Renal Trauma Grade**	**n (%)**	**Patients with reduction volume in CT**	**Average flow reduction in MRA**
III	13 (41.9)	23,1 %	42,7 %
IV p	9 (29)	44.4 %	34.5 %
IV v	7 (22.6)	85.7 %	50.1 %
V	2 (6.5)	100 %	86.5 %

**Figure 1:**
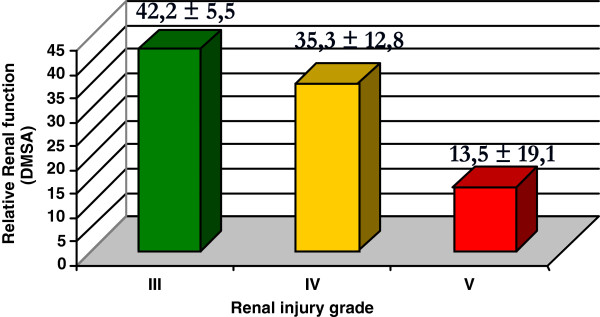
Distribution of the relative renal function expressed as absolute values according to the severity of the renal trauma.

The DMSA renal scintigraphy was performed on all the patients. The relative renal function was severely impaired (less than 30% in the injured kidney) in 6 patients (19.4%), of whom 66.7% had had renal trauma grade IV with vascular injury (4 cases). The other patients were the two grade V renal injuries. The relative renal function was moderately impaired (between 30-40% in the injured kidney) in 8 patients (25.8%), with 50% of the cases being grade III (4), 25% grade IV with extravasation (2) and 25% grade IV with pedicle injury (2). The relative renal function was normal to mildly impaired (greater than 40% in the injured kidney) in 15 patients (48.4%). Of these cases, 60% had grade III renal trauma (9), 33.3% grade IV with extravasation (5) and one grade IV case with injured pedicle. Figure 1 shows the functional result of the non-operative treatment of renal trauma through the relative renal function with DMSA expressed as absolute values according to the severity of the trauma.

**Figure 2:**
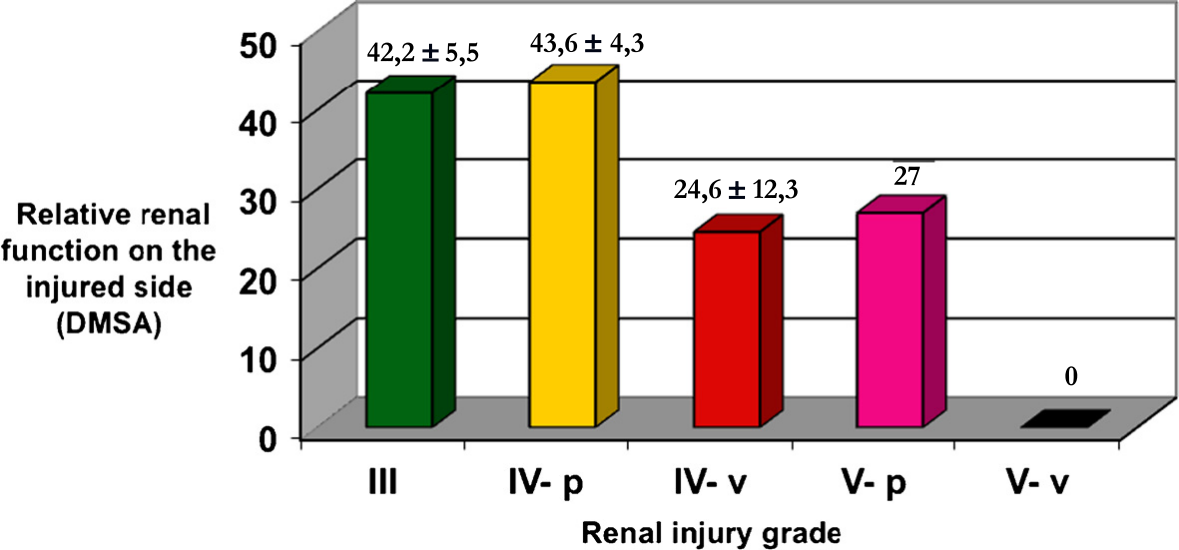
Distribution of the relative renal function expressed as absolute values according to subdivisions of grade IV and V renal traumas (IV - p: with extravasation; IV - v: with pedicle injury; V - p: multiple fractures; V - v: with total ischemia).

The functional results of parenchymal and vascular causes for grades IV and V renal injuries were separately subdivided into: grade IV with extravasation (IV-p), grade IV with pedicle injury (IV-v), grade V with multiple fractures (V-p) and grade V with devascularized kidney (V-v). Figure 2 displays the distribution of the relative renal function expressed as absolute values according to the subdivisions of grade IV and V renal traumas.

Statistical analysis of the relative reductions in renal function of the injured side by group was showed in Table 4. The comparison of the relative renal function of the injured side among the patients of the different grades of renal injury showed significant variation (p < 0.01). For having only two values, the injuries of grade V do not allow comparisons.

**Table 4:** Relative reductions in renal function of the injured side by group

**Comparison among groups**	**Value of p**
Group III x Group IV p	p > 0,05
Group III x Group IV v	p < 0,01
Group IV p x Group IV v	p < 0,01

All patients the blood pressure records during the hospital stay for renal trauma were normal. The use of ambulatory blood-pressure monitoring allowed the identification of 29% of cases of arterial hypertension (9 patients), only one of which was known to be hypertensive. All of whom were male with average age of 35.6 years (22 to 69 years). The average time between the trauma occurrence and the study was 7.8 years, ranging from 1 year and 4 months up to 13 years and 4 months. The trauma mechanism was blunt in 7 (77.8%) of the cases. In relation to the severity of the renal trauma, 6 (66.7%) had grade III, one showed grade IV with urinary extravasation, one had grade IV with pedicle injury and another presented grade V with multiple fractures of renal parenchyma. Paternal and/or maternal familial antecedents of arterial hypertension were found in 7 (77.8%) of the cases. DMSA renal scintigraphy showed two patients with severely impaired relative renal function (< 30%), two with moderate impairment (between 30 and 40%) and 5 cases with normal to mild impairment on the injured side (> 40%).

Dynamic renal scintigraphy was performed on 7 of the 9 hypertensive patients. The examination was not performed on the other two since their relative renal function on the injured side was less than 25%, which would not allow a conclusive result. None of the studied patients presented alterations of the captation curves after sensitization with captopril, based on a negative test result.

## Discussion

Several studies have demonstrated the success of the non-operative management of renal injuries, indicating that the decision concerning the expectant or surgical management does not have to be made based only on the grade of the tomographic staging of the injury, but also by taking into consideration the clinical picture, the hemodynamic state, the presence of associated injuries and the blood transfusion requirements [2,3,27-30].

The reduction of the renal volume observed by computed tomography in 50% of the patients and the percentage of renal volume reduction were found to be related to renal trauma severity as defined by OIS, including the subdivisions of grades IV and V. Our results confirm that the degree of renovascular injury and the extent of nonperfusion of the kidney at admission CT scan appear to determine the functioning volume loss observed by nuclear scanning at the follow-up assessment was highlighted by previous series [1,10].

Functional studies of the kidneys, like angiography and flow measurements, using MR imaging were not possible until recently, because motion from respiratory cycle and perturbation of magnetic field, near the interface between gas within bowels and pericolonic fat interfere with data acquisition. The sensitivity and specificity in the detection of significant renal stenosis (> 50%) are 100% and 93%, respectively [23-26]. In this study MR imaging, no renal artery stenosis was founded. Although the asymmetry between the blood flow in both kidneys was detected in most cases, there was no significant difference among the different grades of renal trauma.

DMSA renal scintigraphy is the standard procedure for estimating the functional renal mass because its yields high quality static images of the renal cortex [31,32]. Other series showed that non-operative treatment of renal trauma, specifically in more advanced grades, can be safe with low index of complications and the correlation between AAST grade and relative renal function [1,12-14]. These findings are closed to our results (Figure 1). However, with the subdivisions of the functional outcome of the renal traumas grades IV and V, differentiating the vascular and parenchymal injuries, revealed that the renal injuries of grade III had produced the same functional outcomes as those of grade IV with extravasation (Figure 2). The vascular renal injury grades IV had a significantly worse functional result than those of grades III and IV with extravasation (Table 4). This finding is in disaccording with another previous study [13]. Additional analysis of a larger sample size from multiple institutions should be performed to validate these findings.

Dugi et al even proposed a subclassification of grade IV renal trauma to help decide between non operative management (grade 4a – low risk) and early surgery or angiographic embolization (grade 4b – high risk) based on the presence or absence of a series of important radiographic risk factors, including perirenal hematoma, intravascular contrast extrasavation and renal laceration complexity [33].

This discussion is in accordance with the revision proposed to updated the AAST OIS for renal trauma [34]. Actually, the classification is based primarily on parenchymal laceration depth and the presence or absence of vascular injury [33]. It is necessary this revision to eliminate existing confusion and inaccurate renal staging by creating a precise and complete renal staging classification to guide clinical management and to facilitate renal trauma research, particularly in grades IV and V [34]. Also, the functional outcome of renal trauma based on the initial radiological evaluation would help us avoid multiple time and cost consuming procedures to salvage a nonfunctional kidney [14].

Future alterations in the current classification of renal injury gravity would be advanced by imaging diagnostic methods that would allow the identification of extravasation of contrast in arterial segments, quantitative measures of the volume of the hematoma and other variables that would predict, in a more precise manner, the results of renal trauma [29]. Information about evaluation of renal function after trauma could be included in revision of AAST providing additional strength to the injury scale as an instrument to predict clinical outcomes after renal trauma.

The complications that may arise from non-operative management of renal trauma include: urinoma, perinephritic abscess, delayed hemorrhage and arterial hypertension [29,30].

Some authors who assessed the incidence and prevalence of post-traumatic renal hypertension [35-41], with different times of follow-up, have commented on the factors related to the etiology of arterial hypertension [19,42-45].

Monstrey et al [19]., who studied 622 patients with renal trauma to evaluate the incidence and prevalence of posttraumatic arterial hypertension, did not observe any increase in the incidence of arterial hypertension. They found no definitive relation between hypertension and renal trauma. In the same article, the authors reviewed 71 publications from the medical literature which showed that more than 90% of 223 cases previously described as posttraumatic renal hypertension, in fact did not confirm with the diagnosis due to the following reasons: documented normal blood pressure before the injury, associated renal disease, non-validated hypertension, no definition of the anatomical injury or its functional significance. Most documented cases can be classified into one of three types of renal lesions known to produce renal ischemia with subsequent development of hypertension, namely, renal artery stenosis (Goldblatt mechanism) [42], external renal compression (Page mechanism) [43], and intra-renal arteriovenous fistula [44]. In this study, none of these types of damage was founded in imaging evaluation of posttraumatic renal injuries.

The diagnostic refinement derived from the use of ambulatory blood-pressure monitoring allowed the identification of 29% of cases of arterial hypertension (9 patients). No previous study in the literature on renal trauma and arterial hypertension had used ambulatory blood pressure monitoring. It is important to note the low average age of the hypertensive patients with future cardiovascular risks associated with the high rate of familial arterial hypertension. There was no direct correlation between the grade of renal injury and the presence of arterial hypertension, although 66.7% of the cases had renal injury of grade III. Morphological evaluation by both computed tomography and magnetic resonance angiography excluded any possibility of renal artery stenosis, external renal compression or arteriovenous fistula. Furthermore, there was no correlation between a serious reduction of renal function found by DMSA renal scintigraphy and the presence of arterial hypertension.

In the patients with renovascular hypertension, the dynamic renal scintigraphy with the use of the ^99m^Tc EC demonstrates a gradual accumulation of the radionuclide in the kidney affected during the phase of the study after captopril administration. This can be explained by the reduced glomerular filtration rate, measured scintigraphically as delayed uptake and cortical retention. Investigators have reported the test to have approximately 90% sensitivity and more than 95% specificity [31,46].

The diagnosis of a rennin-dependent renovascular hypertension was excluded in all patients, suggesting that arterial hypertension may be essential.

## Conclusions

The present study showed that non-operative management of renal trauma, specifically in high grades, can be safe with low index of complications. The late functional outcome was favorable in patients with renal injuries of grades III and IV with extravasation, differing significantly from the worse functional outcome in those of grades IV and V with vascular injuries, suggesting that the degree of renovascular injury and the extent of nonperfusion of the kidney at admission CT scan appear to determine the functioning volume loss observed by abdominal CT scanning at the follow-up assessment. The long-term follow-up showed 29% of the patients were hypertensive without direct correlation with the grade of renal injury or serious reduction of renal function found by DMSA renal scintigraphy. Renal etiology of arterial hypertension could be excluded by dynamic renal scintigraphy with the use of the ^99m^Tc EC with captopril-stimulated study, suggesting that posttraumatic arterial hypertension can be essential. A revision of AAST renal trauma is necessary to correct the inconsistent in the definition of a grade IV and V renal injury making discussion of management and comparison of outcomes difficult and not reliable. There are news knowledge involving management of renal trauma derived from clinical experience, research, precise radiographic staging, renal function studies and new innovation and technology that can be incorporated into a revision of current classification.

## Competing interests

The authors declare that they have no competing interests.

## Authors’ contributions

Study Design: PJ, M, S Data Collection/Analysis/Interpretation: PJ, M, S, N, K, N Manuscript Drafting: PJ, M, A Critical Review: M, N, S. All authors read and approved the final manuscript.
